# COVID-19 pandemic in India: A Comparison of pandemic pattern in Selected States

**DOI:** 10.3126/nje.v10i2.28960

**Published:** 2020-06-30

**Authors:** Rama Shankar Rath, Anand Mohan Dixit, Anil Ramesh Koparkar, Pradip Kharya, Hari Shanker Joshi

**Affiliations:** 1 Assistant Professor, Department of Community Medicine & Family Medicine, All India Institute of Medical Sciences, Gorakhpur; 2-5 Department of Community Medicine & Family Medicine, All India Institute of Medical Sciences, Gorakhpur

**Keywords:** Coronavirus, COVID-19, Mortality, India, SARS-COV-2, quarantine

## Abstract

The COVID-19 pandemic currently expanded its roots to the 206 countries in the world. The morbidity and mortality are not only threat to humans but also its impact on economy is indirectly affecting us. The current review was done to find trend in various states of India. Data was collected from Ministry of Health and Family Welfare and descriptive analysis of the distribution of COVID-19 cases in different states of India. First case of COVID-19 was diagnosed in southernmost state Kerala and after that it has spread to all other states, but situations are more worsen in states with high international migration. Maharashtra is now the most affected state followed by Delhi. Among epidemic curve of all these states, Maharashtra has rapidly growing epidemic curve with highest slope, whereas Kerala has the lowest. When we compared the day wise cumulative case fatality rate, it was found that the case fatality rate of the states like Maharashtra, Madhya Pradesh & Rajasthan showed decrease in the case fatality rate over the period. Population density is also one of the key determinants of social interaction and thus the spread of disease specifically in communicable diseases. Government of India had taken many strong initiatives e.g. 40 days nation-wide lockdown, thermal screening at airport, announcement of relief packages for poor and quarantine of outsiders but still there are many missed opportunities like, early stoppage of international traffic, compulsory quarantine for all international travellers, better contact tracing, strong law and order and better preparedness plan.

## Introduction

First case of Novel Corona Virus Disease (COVID-19) was reported in December 2019 in Wuhan city of China [1]. Since then, the disease has caused significant concern in the world due to its pestilent nature. Novel Corona Virus pandemic has currently affected more than 200 countries. The disease is not only affecting health of the people but also affecting wealth of concern nation [2].

As of 20thJune 2020, a total of 85,25,042 confirmed cases and 4,56,973 deaths had taken place with no respite in geographical spread, mortality, morbidity, and economic loss due to the virus [3]. Among all the countries highest case load was reported in United States of America (USA) i.e. around 20,57,838 cases followed by, Brazil with 8,50,514 cases and Russian Federation with 5,37,210 cases [3]. India is 4th in the list with reported 3,32,424 cases and 9,520 deaths from novel coronavirus as of 15th June 2020, according to the Ministry of Health and Family Welfare, Government of India [4]. It has been considered that poverty and underdeveloped community are generally predispose to infectious diseases [5]. But persistent and rapid spread of infection of COVID-19 in developed countries with the damage caused to it has also shown the world that developed world are also not immune to the disease. Damage caused by COVID-19 is not only in the physical health but also psychological and socioeconomic in nature.

However, a different pattern of the spread of the pandemic was observed in between the countries and within the country also [6]. Various inherent cultural and social differences might have resulted the same [7]. Similarly, public health policies also has some impact on the spread of the disease [8-10]. As health is a state subject, states within the country are free to take steps that is required to contain the pandemic spread over and above the policy of the central government [11]. Thus, the review was planned with the aim to compare the trend of the pandemic in different Indian States in early phases of the pandemic in India (till 15th June) and to assess the various possible factors for the same.

## Methodology

This is a descriptive analysis of the distribution of COVID-19 cases in different states in India. Data was collected from the official website of Ministry of Health and Family Welfare (MOHFW), Government of India(GOI) on daily basis to extract the number of daily cases, recovered cases and deaths from which the total active cases were calculated [12].

### Analysis:

Data was entered in Microsoft excel 2019 and analysis was done using both MS Excel and STATA 12. Cumulative Case load was calculated from daily case load. Case fatality rate was calculated state wise from the total cases and deaths reported in the same period and recovery rate was calculated. Cumulative case load was plotted against cumulative death to determine the date wise case fatality. Heat map of India was prepared with the help of the Microsoft Excel and Bing Maps.

#### Operational Definition:

1. High Case Load States: Those states which reported more than 300 cases (arbitrarily selected) till 20/04/2020 were followed till 15/06/2020.
2. Day zero: When the state reported the 1st case of COVID-19. Defined as reported by the MOHFW, GOI.
3. Recovery rate: Total number of people reported to be recovered from the disease after the start of the epidemic divided by total number of cases reported by the state in the same period.
4. Case fatality rate: Total number deaths reported by the states divided by the total cases of COVID-19 in the same period. Literature searches

## Results

### Distribution of cases in the Country:

**Figure-1:**
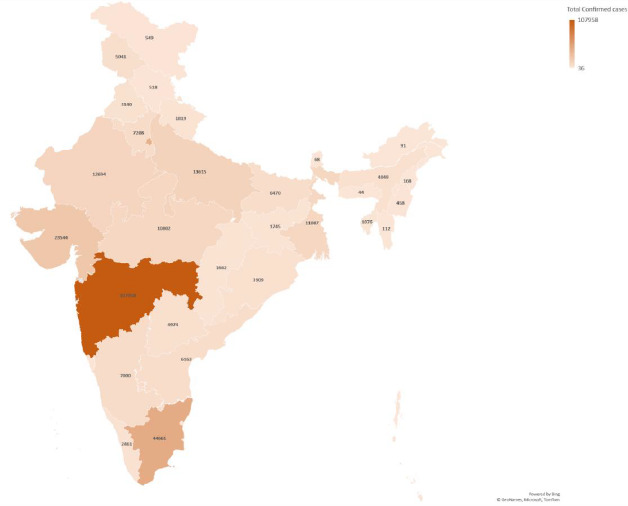
Map Showing Case load of COVID-19 state wise*

India till now (15/06/2020) has reported 3,32,424 number of cases in a span of 132 days after the start of the pandemic in the country on 30/01/2020. States like Maharashtra, Delhi, Tamil Nadu, Madhya Pradesh, Gujarat, Rajasthan & Uttar Pradesh accounts for around 88.5% of case load in the country. Every state and union territory of India is currently affected by the pandemic till now although very few cases were reported in North eastern states like Meghalaya, Mizoram, Nagaland and union territories like Andaman Nicobar islands, Dadra and Nagar Haveli and Daman Diu [Figure-1]. Highest burden of the cases was reported from Maharashtra (107958) followed by Tamil Nadu (44661) and Delhi (41182). Out of the total cases approximately 51% were either cured from disease or migrated whereas a total of 9520 deaths were reported leading to a case fatality rate of 2.9%.

### Onset of the Pandemic in the selected states:

Onset of cases started in India from the last week of January, Kerala being the 1st state to report cases of COVID-19. Kerala also reported the 2nd and 3rd case of COVID-19 in 1st week of February. After a gap of nearly one-month Delhi reported the 4th case of India & its first case in the 1st week of March along with Telangana. Rajasthan Reported the 5th case of the country and 1st case of the state on 3rd March 2020. After this daily case were reported in the affected states. Uttar Pradesh reported 6 cases on 04/03/2020 and Haryana reported 14 cases on same day, highest reported in the country till date. Rest of the high case load states reported the cases after 10th of march.

**Figure-2:**
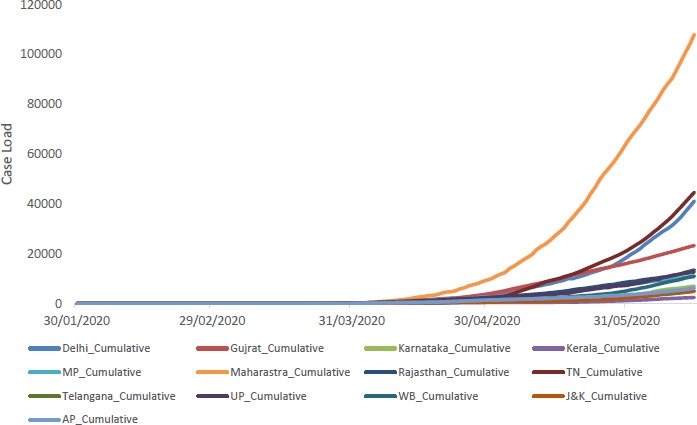
Cumulative Cases in Selected States

When we see the epidemic curve of all these states, Maharashtra has rapidly growing epidemic curve with highest slope followed by Tamil Nadu, Delhi, and Gujarat, whereas Kerala has the lowest slope of the epidemic curve although pandemic has started in each state on different days. [Figure-2]

**Figure-3:**
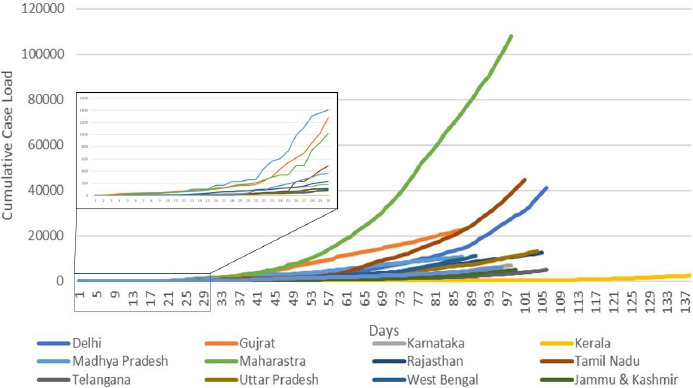
Day wise comparison of cases in Selected States

Since the pandemic in different states started in different days, we tried to analyse the epidemic curve starting from day zero of each state i.e. from the day of reporting of cases by the state. We found that from all the states Madhya Pradesh has the sudden increase in slope starting from day 21 followed by Gujrat which showed a rise on day 23 followed by Maharashtra on day 25th. Kerala showed a long phase of slow growing epidemic followed by a slow rise in identified cases from the 50th day of the epidemic. Rest of the high burden states showed a slope which is in between the slope of Kerala and Maharashtra. [Figure-3] However in the later stage i.e. after 33rd day the case load in Maharashtra started growing at a rapid rate than all other states. Cases in states like Tamil Nadu and in the later stage i.e. around 60th day of the pandemic of the state. Rest all the states have shown a late rise in the case load i.e. around 75th day of the pandemic. Kerala continue to have a slow growing epidemic till now.

Comparison of Outcome of the Patients in different states:

Among the selected states Gujarat reported highest case fatality rate around 6.3% followed by West Bengal (4.3%) and Madhya Pradesh (4.2%). Kerala showed the lowest case fatality rate i.e. around 0.8%.

The proportion of cases recovered from the disease is around 75.4% in Rajasthan which is highest in the country, followed by Madhya Pradesh 71.1% and Gujarat which is around 69.4%. Delhi showed the Lowest recovery rate of around 38.4% preceded by Kerala with recovery rate of 44.8%.

When we compared the day wise cumulative case fatality rate day-wise we found that the case fatality rate of Delhi and Tamil Nadu increased over the period, whereas Maharashtra, Gujarat, Jammu & Kashmir and Uttar Pradesh maintained a constant death rate over the period. Death rate of Madhya Pradesh and West Bengal decreased over the period. For rest of the states due to very low number of cases and death it is very difficult to comment on the case fatality rate over the period.

## Discussion

### Case Load in Different States:

Almost 90% of cases from India is reported from states like Maharashtra, Gujarat, Madhya Pradesh, Delhi, Karnataka, and West Bengal. The reason for higher number of cases in these states can be due to the following:

### International Traffic:

States reporting highest number of cases were mainly those with in and out migration. According to the report of Airport Authority of India, Delhi (27.2%), Mumbai (22.2%), Chennai (9.7%), Cochin (7%), Bangalore (5.7%), Hyderabad (5.2%), Calicut (4.7%), Trivandrum (4.2%), Kolkata (3.8%), Ahmedabad (2.1%) & Trichy (1.9%) shares almost 90% of the total international passenger traffic of India [13]. This may very well explain the higher case load in states with this international traffic.

According to a report of ministry of tourism from all the foreign tourist arrivals in 2019, UK accounts for 10.8% followed by USA (10.5%) Australia (3.6%), Germany (3.16%) and China (3.0%) [14].This may explain the import of cases to India continued till 18/03/2020 when India imposed ban on landing of international flights.

### Migration:

Migration both inter-state and intra-state, is important determinant of transmission of disease across the state and district [15-17]. According to census 2011 report, almost 455 million people i.e. 30% of the total population of India are migrants, from which 15 million has the migration status of less than one year, and 65 million population migrated within 1-4 years where as rest migrated more than five years. Highest out migration is reported from states like Uttar Pradesh (23.0%), Bihar (14.1%), Rajasthan (7.0%), Madhya Pradesh (5.4%), Maharashtra (5.3%), Karnataka (4.7%) [18]. Except Bihar all other states reported the high burden of COVID-19 cases. High burden states also have high rate of interstate migration [18]. Higher rate of migration was also observed between states like Delhi, UP, MP and Maharashtra. This fact is also supported by the reporting of most of the cases in the age group of 25-59 years which is also the age group of the migrant population in India [19]. With the migration of labour from other states, states like Telangana has shown increase in number of cases in the earlier part of May 2020, approximately 1 month after the lockdown was announced. However, there was no reported rapid increase in cases in Uttar Pradesh which can be attributed to the porous border between Delhi and UP which always remained open for migrant workers moving to the home state by foot.

### Population Density:

Population density is also one of the key determinants of social interaction and thus the spread of disease specifically in communicable diseases [20]. In all the high caseload states not all the districts are reporting extremely high number of cases. Generally, cases are reported from very few districts. Based on this fact, Govt. of India have produced a list of 123 hot spot districts with large outbreaks [21]. From these, 109 districts were from high burden states and 55 districts (50%) has population density more than 500 [22]. Similarly, the large outbreaks were reported in the cities like Mumbai, Delhi, Kolkata and Chennai accounting to around one lakh cases in the countries. These cities were also reported to have high population density according to census 2011 [23].

### Progression of Pandemic in selected states of India:

Initiation of pandemic depended up on the in-migration but the progression of pandemic generally depends up on different strategies adopted by the states to stop the transmission. Different rate of rise in cases in the state may be due to the stringency of the actions taken by the state or innovations done at the state and local level. In the early phase of the pandemic various religious travel, travel by the stranded foreign travellers from one part of the country to other part also led to seeding of cases from one part of the country to other part of the country [24]. Among all the states included in the study Kerala reported to have very slow rise in cases. The early control of cases in Kerala may be due to early response of the government of Kerala and readiness of epidemic preparedness. 1st response to pandemic started on 5th January 2020; 25 days prior to the 1st reported case in the state. The following measures that might have favoured Kerala in achieving early slowdown of the case load [25].

1. Aggressive testing of all travellers from China and notified countries
2. Intense contact tracing in last 28 days and follow up
3. Sampling collection centres at the district and local level
4. Early & continued Community involvement
5. Quarantine period of 28 days (as compared to 14 days in rest of the country)
6. Isolation centres near the community
7. Better Hospital Preparedness to prevent spread of infection to health care provider and other patients

None of the states implemented interstate and intrastate travel restriction which led to continuous migration of cases within the states and districts until lockdown announced by the central government [26]. After lockdown also migration of the labour by foot and in the later stage state and central level efforts transport the migrants to the own state had made the condition still worse [27, 28]. However, control of number of cases in Kerala may be due to exposure of the state to the similar epidemic threats in the past in addition to the above-mentioned points [29]. Rise in case load in the different states with different rate may be due to difference in number of tests conducted according to the contact history. States with higher number of tests as a result if the better contact tracing might have detected a greater number of cases whereas states with a smaller number of tests might not be able to detect the hidden cases in the community.

### Outcome of Patients:

Overall, the recovered cases are highest in Rajasthan followed by Madhya Pradesh and Gujarat. Combination of different factors may be responsible for the high cure rate in any geographic area. The first being the process of defining the cure. Government of India changed discharge policy recently has shown increased cured patients in the later part of the May 2020 [30, 31]. The higher proportion of recovered cases in the states like Rajasthan and Gujarat may be due to decrease in the case load in the later part of May. States like Kerala which was showing remarkably high cure rate till the month of April, the cure rate again decreased in the later part of the study period (i.e. after 30th April) may be attributed to the increase in the number of cases. Other states showing a low recovery rate may be due to recent rapid rise in case load in the state. All these factors mixed with the natural history (i.e. certain amount of time required for clearing the infection from the body) of disease might resulted in this pattern of the recovery rate.

While the case fatality rate depends the severity with which cases may be admitted with the hospital, age and health status of severely ill patients (elderly with chronic diseases e.g. Hypertension, Diabetes, chronic kidney diseases are at higher risk of death) and saturation of the health system [32]. States like Delhi might be showing increased case fatality rate due to this reason. Overall, the death rate in many states like Gujarat, West Bengal, Telangana, Maharashtra, and Madhya Pradesh reported death rates higher than that of the country average. Factors like virulence of the virus may be another determining factor that can result in the less death rate in many other states [33]. Higher case fatality rate may also be an indication of hidden cases in the community. It may be due to poor contact tracing inadequate travel history and a smaller number of tests. Although there is no adequate number of samples defined that need to be tested for a population, but higher number of tests may be able to eliminate such artificial bias developed in interpretation of results.

Very few states (West Bengal and Madhya Pradesh) has shown the decrease in case fatality rate over the period. This is a normal phenomenon in an epidemic, as the number of tests increases gradually over the period the denominator becomes large, whereas the numerator remains more or less the same as more severe cases were tested throughout the epidemic period according ICMR guidelines [34, 35]. Similarly, with increase in the testing capacity we are likely to pick a case in the early phase of the disease and treat the same. There also comes the role of treatment states which tried various treatment modalities which led to the cure of the patient in early phases of the disease. Whereas in the scenario of increased testing, the increase in case fatality rate may indicate saturation of the health system and spread of infection in elderly and those with comorbid conditions. This might be the scenario in states like Delhi and Tamil Nadu where the death rate stated increasing recently. Similarly, increased burden of number of patients over the time in the health care facility may add on to the misery of poor doctor patient ratio [36]. However, this statement can be stated with relatively high caution without looking for the ground reality and other unidentified factors.

## Conclusion

Covid-19 is rapidly spreading pandemic disease in India. There are Interstate differences in starting and spread of disease, initiatives for control of the disease and caseload, recovery, and case fatality rate. Few states like Maharashtra, Gujarat, Madhya Pradesh, Delhi, Karnataka, Uttar Pradesh, Rajasthan accounted for almost 90 percent of cases in India. Case load varied with International traffic, inter and intra state migration, population density of the state and districts. State like Kerala have shown better preparedness and better initiatives, resulted in better control over disease initially. Case fatality and recovery rate are varying during progress of pandemic and will depend on population composition, number of investigations done to identify cases, including strategy for contact tracing and status of health services. Though Government had taken many strong initiatives e.g. nation-wide lockdown, announcement of relief packages for poor; there are many missed opportunities like, early stoppage of international traffic, compulsory quarantine for all international travellers, better contact tracing, strong law and order, lockdown in phase 1 of transmission in country and better preparedness plan.

## Figures and Tables

**Figure-4:**
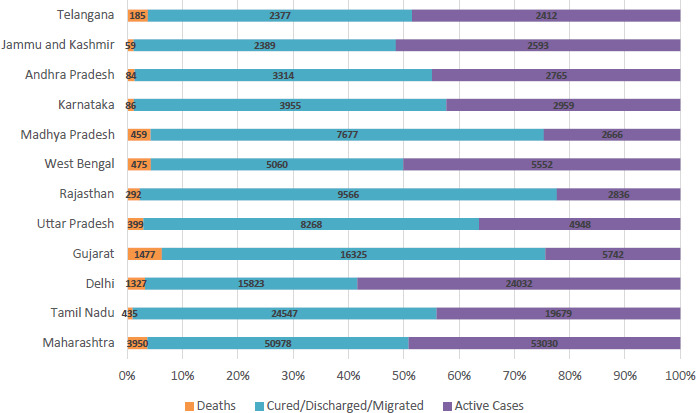
Outcome of COVID-19 cases in selected states

**Figure-5:**
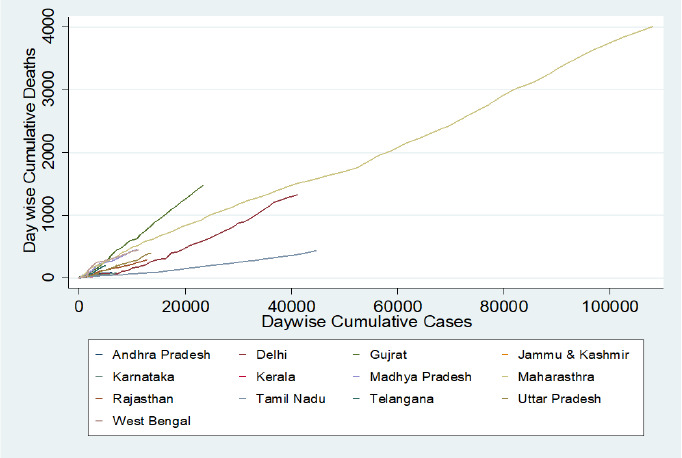
Case fatality rate in selected states during the pandemic
